# Supplementary material for: Utility of Initial Arterial Blood Gas in Neuromuscular versus Non-Neuromuscular Acute Respiratory Failure in Intensive Care Unit Patients
Source: J Clin Med. 2022 Aug 22;11(16):4926. doi: 10.3390/jcm11164926 (PMC9410118; doi:10.3390/jcm11164926)
Supplement: Supplementary file 1 [file jcm-11-04926-s001.zip › jcm-1839699-supplementary.pdf]

Supplementary Materials

Table S1. Prevalence of ABG abnormalities per categories of Respiratory failure
Table S2. Prevalence of ABG abnormalities per disease
Table S3. Sensitivity analysis comparing severe NMRF versus severe non-NMRF and its categories
Table S4. Sensitivity analysis combined blood gases (arterial and venous), comparing NMRF versus non-NMRF and its categories
Table S5. Comparison of ABG data among the different centers in NMRF cases
Figure S1. Scatter plot for ABG parameters data for NMRF and non-NMRF cases.

Table S1. prevalence of ABG abnormalities per categories of Respiratory failure.

	Acidosis	*p* Value *	Hyper-carbia	*p* Value*	Hypoxia	*p* Value *	Elevated Bicar-bonate	*p* Value *	Hypercarbia & Hypoxia	*p* Value*	Isolated Bicarbonate	*p* Value *	Hypercarbia or Hypoxia	*p* Value *
NMRF, n/N (%)	22/69 (31.9)		17/69 (24.6)		23/68 (33.8)		53/69 (76.8)		9/68 (13.2)		23/68 (33.8)		31/68 (45.6)	
Non-NMRF, n/N (%)	120/218 (55.1)	0.001	87/218 (39.9)	0.02	109/216 (50.5)	0.018	129/218 (59.1)	.010	49/216 (22.7)	0.12	32/216 (14.8)	0.001	145/216 (67.1)	0.002
PPD, n/N (%)	31/46 (67.4)	0.000	31/46 (67.4)	0.00	23/45 (51.1)	0.08	34/46 (73.9)	0.825	17/45 (37.8)	0.003	4/45 (8.9)	0.003	36/45 (80)	<0.001
Pneumonia, n/N (%)	45/92 (48.9)	0.036	28/92 (30.4)	0.48	54/92 (58.7)	0.002	54/92 (58.7)	0.019	19/92 (20.7)	0.29	14/92 (15.2)	0.008	63/92 (68.5)	0.006
Pulm. Edema, n/N (%)	36/68 (52.9)	0.016	22/68 (32.3)	0.35	28/67 (41.8)	0.37	30/68 (44.1)	0.000	11/67 (16.4)	0.64	11/67 (16.4)	0.03	38/67 (56.7)	0.23
Others, n/N (%)	8/12 (66.7)	0.048	6/12 (50)	0.09	4/12 (33.33)	1.00	11/12 (91.7)	0.444	2/12 (16.7)	0.67	3/12 (25)	0.74	8/12 (66.7)	0.22

* *p* value with Fisher exact tests, compared to NMRF: neuromuscular respiratory failure.

Table S2. prevalence of ABG abnormalities per disease.

	Acidosis	Hypercar-bia	Hypoxia	Elevated Bicarbonate	Hypercar-bia and Hypoxia	Isolated High Bicarbonate	Hypercar-bia or Hypoxia
Myasthenia gravis, *n* (%)	9/29 (31.0)	3/29 (10.3)	8/28 (28.6)	19/29 (65.5)	3/28 (10.7)	10/28 (35.71)	8/28 (28.6)
Guillain-Barre syndrome, *n* (%)	6/23 (26.1)	6/23 (26.1)	7/23 (30.4)	19/23 (82.6)	2/23 (8.7)	8/23 (34.8)	11/23 (47.8)
Amyotrophic lateral sclerosis, *n* (%)	7/17 (41.1)	8/17 (47.1)	8/17 (47.1)	15/17 (88.2)	4/17 (23.5)	5/17 (29.4)	12/17 (70.6)
Pneumonia, *n* (%)	45/92 (48.9)	28/92 (30.4)	54/92 (58.7)	54/92 (58.7)	19/92 (20.7)	14/92 (15.2)	63/92 (68.5)
COPD, *n* (%)	19/29 (65.5)	22/29 (75.8)	17/28 (60.7)	24/29 (82.8)	13/28 (46.4)	2/28 (7.1)	25/28 (89.3)
Asthma, *n* (%)	8/12 (66.7)	4/12 (33.3)	3/12 (25)	5/12 (41.7)	1/12 (8.3)	2/12 (16.7)	6/12 (50)
Bronchiectasis, *n* (%)	4/5 (80.0)	5 (100)	3/5 (60.0)	5/5 (100)	3/5 (60)	0/5 (0.0)	5/5 (100)
Heart failure, *n* (%)	28/56 (50.0)	19/56 (33.9)	25/55 (45.5)	27/56 (48.2)	9/55 (16.4)	10/55 (18.2)	34/55 (61.8)
Non cardiac Pulmonary edema, *n* (%)	8/12 (66.7)	3/12 (25)	3/12 (25.0)	3/12 (25)	2/12 (16.7)	1/12 (8.3)	4/12 (33.3)
Others, *n* (%)	8/12 (66.7)	6/12 (50.0)	4/12 (33.3)	11/12 (91.7)	2/12 (16.7)	3/12 (25)	8/12 (66.7)

* *p* value with Fisher exact tests, compared to NMRF: neuromuscular respiratory failure.

Table S3. Sensitivity analysis comparing severe NMRF versus severe non-NMRF.

	pH	*p* Value *	PaCO2	*p* Value *	PaO2	*p* Value*	HCO3	*p* Value *
Neuromuscular respiratory failure, median (IQR)	7.38 (7.3–7.42)		42.9 (35.7–54.15)		87 (74.8–124)		24.7 (22.7–29.1)	
Non-Neuromuscular respiratory failure, median (IQR)	7.32 (7.2–7.41)	0.01	44.1 (35.8–65.35)	0.40	77.3 (63.4–96.2)	0.004	23.1 (18.85–26.75)	0.01
Pulmonary disease (COPD, asthma and Bronchectasis)	7.28 (7.15–7.41)	0.03	65.6 (49–81)	0.001	79.9 (64.9–88.2)	0.09	29.65 (22.1–32.8)	0.25
Pneumonia, median (IQR)	7.325 (7.22–7.41)	0.059	41.75 (35.7–62.3)	0.97	74.5 (62.4–93.3)	0.003	23.2 (18.9–26.5)	0.02
Heart failure and pulmonary edema, median (IQR)	7.31 (7.17–7.40)	0.02	44.55 (32.9–59.4)	0.79	80.6 (60.4–101)	0.13	21.2 (17–25.3)	0.002
Others, median (IQR)	7.34 (7.29–7.4)	0.62	51.6 (35.5–62.2)	0.67	83.1 (80–103)	0.6	25.4 (22.7–26)	0.57

* *p* value, Wilcoxon rank-sum (Mann-Whitney) test, compared to NMRF (neuromuscular respiratory failure). IQR: interquartile range.

Table S4. Sensitivity analysis combined blood gases (arterial and venous), comparing NMRF versus NNMRF and its categories.

	pH	*p* Value *	PaCO2	*p* Value*	PaO2	*p* Value *	HCO3	*p* Value *
Neuromuscular respiratory failure, median (IQR)	7.39 (7.34–7.42)		39.2 (35.3–48.2)		87 (70.8–122.7)		24.35 (22.5–27.1)	
Non Neuromuscular respiratory failure, median (IQR)	7.33 (7.23–7.39)	0.00	43.5 (35.7–62)	0.10	79.8 (64.6–99.6)	0.02	23.4 (19.4–26.7)	0.01
Pulmonary disease (COPD, asthma and Bronchiectasis)	7.29 (7.2–7.36)	0.00	64.3 (46–77.25)	0.00	80.7 (69.8–105)	0.21	27.45 (22.45–29.65)	0.24
Pneumonia, median (IQR)	7.36 (7.27–7.41)	0.02	40.75 (35.2–55.1)	0.89	74.5 (61.1–96.3)	0.003	23.4 (19.6–25.5)	0.01
Pulmonary edema, median (IQR)	7.33 (7.22–7.38)	0.00	41.9 (33.1–53.1)	0.92	86.6 (64.6–101)	0.25	20.9 (17.3–25.5)	0.00
Others, median (IQR)	7.34 (7.3–7.39)	0.14	49.5 (36.6–64.1)	0.24	83.5 (76.7–101.1)	0.65	24.15 (22.5–26.1)	0.7

* *p* value, Wilcoxon rank-sum (Mann-Whitney) test compared to NMRF (neuromuscular respiratory failure). IQR: interquartile range.

Table S5. Comparison of ABG data among the different centers in NMRF cases.

	pH	*p* Value*	PaCO2	*p* Value*	PaO2	*p* Value*	HCO3	*p* Value*
KAUH, median (IQR)	7.39 (7.29–7.48)	0.43	41.5 (34.6–57.5)	0.79	82.5 (66.65–120.5)	0.39	24.7 (23–27.8)	0.88
Other centers, median (IQR)	7.39 (7.34–7.41)		41.5 (36–48.3)		87.5 (87.5–123.4)		25 (22.9–28)	

KAUH: king Abdulaziz University hospital. * Wilcoxon rank-sum (Mann-Whitney) test.

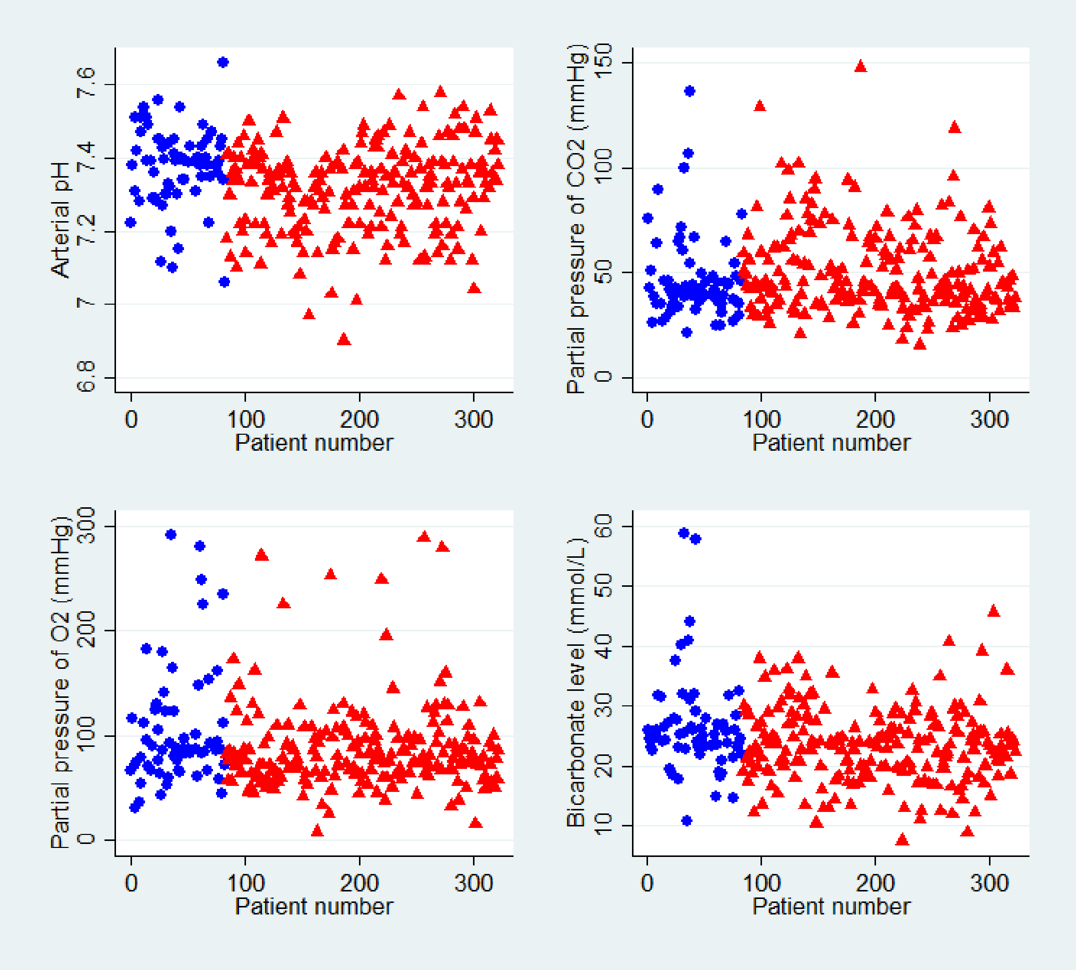

Figure S1. Scatter plot for ABG parameters data for NMRF and non-NMRF cases. Blue circles = NMRF (neuromuscular respiratory failure). Red triangle = Non-NMRF (non- neuromuscular respiratory failure). * note: The purpose of the graphs is to visualize the distinction between NMRF and non-NMRF and hence 1 or 2 outliers were removed from the graphs in order to avoid stretching the graph and to avoid making the data condensed in a small area.
